# Functional Foods and Nutraceuticals in the Primary Prevention of Cardiovascular Diseases

**DOI:** 10.1155/2012/569486

**Published:** 2012-04-10

**Authors:** Eman M. Alissa, Gordon A. Ferns

**Affiliations:** ^1^Faculty of Medicine, King Abdul Aziz University, P.O. Box 12713, Jeddah 21483, Saudi Arabia; ^2^Faculty of Healthand Institute of Science & Technology in Medicine, Keele University, Staffordshire ST4 7QB, UK

## Abstract

Cardiovascular disease (CVD) is now the leading cause of death globally and is a growing health concern. Dietary factors are important in the pathogenesis of CVD and may to a large degree determine CVD risk, but have been less extensively investigated. Functional foods are those that are thought to have physiological benefits and/or reduce the risk of chronic disease beyond their basic nutritional functions. The food industry has started to market products labelled as “functional foods.” Although many review articles have focused on individual dietary variables as determinants of CVD that can be modified to reduce the risk of CVD, the aim of this current paper was to examine the impact of functional foods in relation to the development and progression of CVD. Epidemiologic studies have demonstrated the association between certain dietary patterns and cardiovascular health. Research into the cardio-protective potential of their dietary components might support the development of functional foods and nutraceuticals. This paper will also compare the effect of individual bioactive dietary compounds with the effect of some dietary patterns in terms of their cardiovascular protection.

## 1. Introduction

Cardiovascular disease (CVD) is now the leading cause of death globally and is a growing health concern [1]. Lifestyle-related conditions, such as obesity, hyperlipidemia, type 2 diabetes, and hypertension, are also widespread and becoming more prevalent globally [2]. Although the traditional cardiovascular risk factors (Table 1) have been extensively investigated, dietary factors are also important in the pathogenesis of CVD and may to a large degree determine CVD risk factors such as blood pressure and dyslipidaemia, but have been less extensively investigated.

The role of dietary factors has been largely investigated by reducing the content of specific food component known to increase risk, that is, sodium or saturated fats [3] or by large longitudinal cohort studies in which baseline dietary intake was related to cardiovascular outcomes [4, 5].

The term “functional foods” has been commonly used in marketing but still lacks a firm regulatory definition [6]. Functional foods are those that are thought to have physiological benefits and/or reduce the risk of chronic disease beyond their basic nutritional functions [7]. The food industry has started to market products with a “functional food” label. Whilst the benefits of some functional food constituents may be perceived to enhance short-term well-being, the benefits are generally related to the long-term mitigation of certain diseases.

Although many review articles have focused on individual dietary variables as major determinants of CVD that can be modified in order to reduce the risk of CVD, the aim of this current paper was to examine the impact of functional foods in relation to the development and progression of CVD. Epidemiologic studies have long demonstrated the association between certain dietary patterns and cardiovascular health. Research into the cardioprotective potential of their dietary components may support the development of functional foods and nutraceuticals. This paper will also compare the effect of individual bioactive dietary compounds with the effect of some dietary patterns in terms of their cardiovascular protection.

## 2. The Protective Effect of Diet in CVD

It has been proposed that CVD can be prevented by lifestyle changes, including diet [8]. Early evidence for the role of diet on CVD came from data on trends in food consumption, and ecological studies has shown associations between CVD prevalence and fat intake [3]. Moreover, excessive consumption of foods that are caloriedense, nutritionally poor, highly processed, and rapidly absorbable can lead to systemic inflammation, reduced insulin sensitivity, and a cluster of metabolic abnormalities, including obesity, hypertension, dyslipidemia, and glucose intolerance [9]. More recently, there has been a focus in nutrition research to try and understand the effects of whole foods [10]. An integrated approach combining lifestyle modification with the correct pharmacologic treatment is sought to reduce cardiovascular risk factors, to improve vascular health, and to reduce healthcare expenditure [11].

Whilst epidemiological studies have identified a relationship between diet and CVD, there is still considerable scientific uncertainty about the relationship between specific dietary components and cardiovascular risk. Observational, prospective cohort studies suggest that a higher dietary intake or supplementation of antioxidants is associated with a lower risk of CVD and mortality, but the evidence from clinical trials is still largely negative [12]. The conflicting results between the apparent protective effects of nutrients as part of dietary intake and the lack of effectiveness of single nutrient supplementation in trials has led to a focus on whole foods or modified diets as protective against CVD [13].

**Figure 1:**
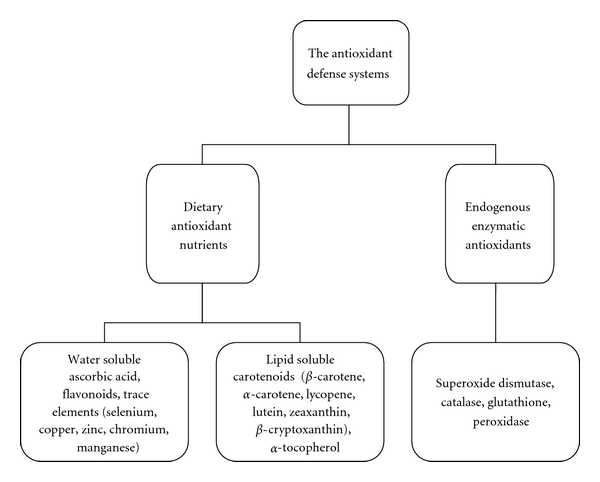
The antioxidant defense system comprise, endogenous enzymatic and exogenous nonenzymatic nutrients. The dietary antioxidant nutrients can either be water soluble or lipid soluble. There are also other dietary constituents that may have either direct antioxidant activity or indirect antioxidant activity such as trace elements that are constituents of antioxidant enzymes.

Oxidative stress may be defined as a disturbance in the pro-oxidant/antioxidant balance that favours oxidation. It has been suggested that oxidative stress is involved in the etiology of several chronic diseases including CVD, diabetes, stroke, some cancers, and neurodegenerative disorders [14]. Dietary nutrients, both water soluble and lipid soluble, comprise an important aspect of the antioxidant defense system (Figure 1).

Beyond their normal occurrence in cells and tissues of living organisms, free radicals and reactive species are also produced in the foods people consume every day, inducing undesirable reactions like oxidation of lipids, proteins, nucleic acids, and carbohydrates.

An impaired capacity to scavenge free radicals and reactive species as a consequence of decreased levels of antioxidant cellular defense systems or excessive free radicals production is common in brain, liver, heart, and other important target organs in humans and animals [15, 16].

It was initially thought that antioxidant vitamins would have represented a simple approach to counteract increased oxidative stress by cardiovascular risk factors. Nonetheless, high doses of some antioxidant vitamins were found to exert adverse vascular effects. The effects of antioxidant supplements on all-cause mortality of adults included in primary and secondary prevention trials, by treatment with beta carotene, vitamin A, and vitamin E, may indeed increase mortality, and the potential role of vitamin C and selenium on mortality needs further study [17, 18].

## 3. Functional Foods

The notion that foods not only provide basic nutrition but can also prevent diseases and ensure good health and longevity is now attained greater prominence [19]. Various terms have been used interchangeably to designate foods for disease prevention and health promotion. The term *Nutraceuticals* was introduced in 1989 by the US Foundation for Innovation in Medicine and referred to “any substance that is a food or a part of a food and provides medical or health benefits, including the prevention and treatment of disease” [20]. The interest in nutraceuticals for cardiovascular prevention was particularly stimulated after the observations of a close association between the consumption of particular dietary factors, as indicated by higher plasma levels [21, 22], and a reduced cardiovascular event rate. In 1994, the US Institute of Medicine's Food and Nutrition Board defined *functional foods* as “any food or food ingredient that may provide a health benefit beyond the traditional nutrients it contains” [6, 7, 23].

## 4. Functional Foods with Health-Related Properties

The conflicting results between the apparent protective effects of nutrients as part of entire food intake and the lack of effectiveness of single nutrient supplementation in trials has led to a focus on whole foods as protective against CVD. Populations consuming a large proportion of plant-based foods, including fruits and vegetables, or those with high intake of seafood are known to have a lower incidence of CVD and certain types of cancer [24]. Based on these findings, considerable interest has been expressed by manufacturers, consumers, and health professionals in functional foods and nutraceuticals.

Functional foods, containing physiologically active components either from plant or animal sources, marketed with the claim of their ability to reduce heart disease risk focusing primarily on established risk factors, that is, blood cholesterol, diabetes, and hypertension. Functional foods are suspected to exert their cardioprotective effects mainly through lipid lowering effects, antioxidant actions, and/or decreased homocysteine levels (Table 2).

Vegetable and fruit fibers (with pectin), garlic and oily seeds (walnut, almonds, etc.), and fish oils have lipid-lowering effects in humans, through both inhibition of fat absorption and suppression of hepatic cholesterol synthesis. Homocysteine increases the risk of both cardiovascular and cerebrovascular disorders [25] by enhancing arteriolar constriction and decreasing endothelial vasodilation [26]. A higher intake of folate, antioxidant vitamins, whole grains, and phytochemicals has been reported to abrogate the deleterious vascular effects of homocysteine in the heart [27, 28]. A significant cardiovascular benefit of phytochemicals (polyphenols in wine, grapes, and teas), vitamins (ascorbate, tocopherol), and minerals (selenium, magnesium) in foods [19, 29] is thought to be the capability of scavenging free radicals produced during atherogenesis.

Several functional foods are thought to be of benefit in treating and preventing CVD. The most common functional foods that have been studied in cardiovascular patients are long-chain n-3 fatty acids, dietary fiber, and phytochemicals as well as nutrients based on or enriched with vegetable proteins, mainly soy.

### 4.1. Fish

People with a high intake of dietary fish and fish oil supplements have a low rate of CVD [30, 31]. Although fish per se contains various nutrients with potentially favourable effects on health, attention has been particularly focused on the omega-3 (n-3) fatty acids. n-3 fatty acids also include the plant-derived alpha-linolenic acid (ALA, 18 : 3 n-3), eicosapentaenoic acid (EPA, 20 : 5 n-3), and docosahexaenoic acid (DHA, 22 : 6 n-3). Both EPA and DHA are found in oily fish, such as salmon, lake trout, tuna, and herring, and fish-derived products (fish oils). n-3 fatty acids precursor, *α*-linolenic acid, is typically found in various plants (e.g., spinach), seeds (nuts and flaxseeds), and oils derived from them. Generally, very little ALA is converted to EPA, and even less to DHA, and therefore direct intake of the latter two is optimal.

Despite the established beneficial effect of fatty fish consumption on CHD, the species and amount of fish consumed, as well as the preparation method, have an impact on CHD risk [32]. The concomitance of low amounts of n-3 fatty acids in our average diet and the need for prolonged administration for prevention and treatment has led to the development of selected preparations (enriched foods or special formulations, e.g., n-3 fatty acids incorporated into cows' milk). These should combine acceptability and adequate bioavailability of their relatively low contents of n-3 fatty acids. These fatty acids are therefore being incorporated into a number of commercially available, natural foods that, due to rather their structural features, appear to be particularly suited as efficient fatty acid vehicles [33]. Furthermore, some environmental contaminants found in certain fish, for example, methylmercury, polychlorinated biphenyls, and dioxins, may diminish the health benefits of fish-derived n-3 fatty acids [34].

Potential mechanisms for the cardiovascular protective effects of n-3 fatty acids are suggested to be; anti-inflammatory, antithrombotic (reduced platelet aggregability), and antiarrhythmic (reducing the risk of potentially fatal cardiac arrhythmias), lowering of heart rate and blood pressure, hypotriglyceridemic, and improved endothelial function [35].

Fish oil supplements have favorable effects on lipid profile and blood pressure [36, 37]. The former appears to be due to decreased hepatic triglyceride secretion combined with enhanced clearance of triglycerides from plasma. Moreover, fish ingestion has been related to a reduced risk for myocardial infarction, which may relate to beneficial effects of EPA and DHA on plaque stability (probably related to the content of inflammatory cells) and modulation of endothelial function [38]. EPA and DHA have also been shown to lower low-density lipoprotein (LDL) oxidative susceptibility in postmenopausal women, which could help to reduce the risk of CVD [39]. Primary among these is the reduction of serum triglycerides [40]. While research interests in the effects of n-3 fatty acids have been focused mainly on the long-chain compounds (EPA and DHA), the role of n-3 ALA should also be considered. Data on the effects of ALA on CVD outcomes are somewhat limited [41, 42], as some researchers suggest potential cardiovascular protection by this unsaturated fatty acid [43].

A meta-analysis of 65 studies demonstrated that n-3 fatty acids lowered triglycerides levels in a dose-dependent manner, with the triglycerides lowering being proportional to baseline levels [44]. However, the data demonstrate a large interstudy variability in response to the treatment that is possibly due to the dose or ratio of n-3 fatty acids, the duration of study, the health status, diet, and other confounding factors. The large heterogeneity within studies with n-3 fatty acids supplementation for the response to triglycerides is likely to be attributable to genetic variability within the study population. Nevertheless, these intervention studies are largely short term and involved frequently higher dose of n-3 fatty acids as compared to the clinical outcome studies. Furthermore, more studies are needed to determine whether there exist specific genotypes that may benefit to a greater extent from n-3 fatty acids for hypotriglycerolaemic effects. Clinical studies also need to determine whether the reduction in CVD risk factors is due to EPA, DHA, or the combination of both and the dosage of the effective components [31].

### 4.2. Fruit and Vegetables

There is a substantial amount of literature that has consistently reported the beneficial effects of diets rich in vegetables and fruits on CVD risk [45–48]. Conversely, inadequate consumption of fruit and vegetables has been linked with higher incidence of CVD [49]. The benefits of fruit and vegetable intake appear to be dose related. In addition, frequency of fruit and vegetable intake has been associated with lower CVD risk [50, 51].

The mechanisms by which fruit and vegetables exert their protective effects are not entirely clear but likely include antioxidant and anti-inflammatory effects. Among the possible explanations for this beneficial effect, fruits and vegetables have been found to decrease susceptibility of LDL particles to oxidation [52]. Potassium may also have a protective role on the incidence of CVD as mounting evidence indicates an inverse association between dietary intake of fruits and vegetables and blood pressure [53, 54]. Several bioactive components in fruits and vegetables such as carotenoids, vitamin C, fiber, magnesium, and potassium act synergistically or antagonistically to promote a holistic beneficial effect. The totality of the evidence supports current dietary guidelines to increase fruit and vegetable consumption to at least five.

Soluble fibres including pectins from apples and citrus fruits, *β*-glucan from oats and barley, and fibres from flaxseed and psyllium are known to lower LDL-C [55]. The mechanisms of their cholesterol-lowering effects are suggested to be the binding of bile acids and inhibition of cholesterol synthesis. However, in the Health Professionals Follow-Up Study, only cereal fiber, not fruit or vegetable fiber, was inversely associated with risk of total stroke [56].

In contrast, several studies have not seen significant protective effects of fruit and vegetables on mortality, although most show protective trends. These include a study of adults in Maryland [57], the Kuopio Ischaemic Heart Disease Risk Factor (KIHD) study among middle-aged Finnish men [58], and the Adventist Health Study [59]. These studies may have had insufficient power, or inadequate ranges of intake to observe significant effects.

### 4.3. Nuts and Legumes

Nuts are complex foods containing cholesterol lowering mono- and polyunsaturated fatty acids, arginine (a precursor to the vasodilator nitric oxide), soluble fiber, and several antioxidant polyphenols [60]. Postprandial vascular reactivity is characterized by decreased bioavailability of nitric oxide and increased expression of pro-inflammatory cytokines and cellular adhesion molecules [61]. It is not surprising that the evidence supporting the cardioprotective effects of diets high in nuts is robust as multiple mechanisms work together to reduce risk. Prospective data from the Physicians' Health Study [62] indicated reduced risk of sudden cardiac death associated with nut consumption originally perceived as being unhealthy because of their high-fat content.

Legumes are also complex foods rich in soluble fibers and polyphenols, as well as folic acid. Legumes were the only food group predictive of survival among five long-lived elderly cohorts in Japan, Sweden, Greece, and Australia [63]. Furthermore, cumulative evidence from experimental research indicates that cholesterol-lowering effect of legumes are probably due to the combined effects of several bioactive components, such as protein, soluble and insoluble fibres, and phytosterols [64]. A recent interventional trial in humans has shown that lupin kernel flour added to bread has also a positive effect on blood pressure: both the fibre and the protein were suggested to be responsible [65].

### 4.4. Whole Grains

Whole grain products contain intact grain kernels rich in fiber and trace nutrients. They are nutritionally more important because they contain phytoprotective substances that might work synergistically to reduce cardiovascular risk.

The potential protective role of whole grains was first evaluated in the early 1970s [66]. Based on the results of the prospective Iowa Women's Health Study that demonstrated cereal fiber had different associations with total mortality, depending on whether the fiber came from foods that contained primarily whole grain or refined grain [67]. A more recent meta-analysis based on seven qualifying prospective cohort studies focused on whole grain consumption and cardiovascular outcomes reported that the inverse association between dietary whole grains and incident CVD was strong and consistent across trials [68–71].

The mechanisms underlying the protective effect of whole grains on CVD risk include its effects on insulin sensitivity [28], blood pressure [72], lipids, and inflammation [73]. Although the anti-inflammatory mechanism is not clear, it may be related to higher intakes of antioxidant nutrients present in the germ of whole grains. As compared to refined grains, whole grains have a reduced glycemic response following ingestion (i.e., the postprandial rise in blood glucose is lessened), and reductions in postprandial glucose surges have been associated with reduced reactive oxygen generation after a meal and reduced postprandial inflammation and CVD risk [28].

### 4.5. Soy Proteins

Soy is the main source of protein in the Japanese diet, consumed in the form of miso soup and tofu. Soy products are rich in polyunsaturated fatty acids, fiber, vitamins and minerals, and low saturated fat content [74].

Prospective observational studies, initially in vegetarians [75], then in Chinese women [76], and in a Japanese population [77], have shown a reduction of total cholesterol and LDL-C as well as of ischaemic and cerebrovascular events with a daily soy protein intake of more than 6 g, compared with less than 0.5 g. A large number of clinical studies were summarized in a meta-analysis [78] and confirmed that serum LDL-C concentrations are modified, the effects being related to baseline blood cholesterol levels. The results of this meta-analysis were criticized recently, since more recent studies appeared not to confirm the very powerful cholesterol-reducing effect of soy proteins [74]. A recent systematic review of available randomized controlled studies mainly in subjects with moderate hypercholesterolaemia confirmed that the inclusion in the diet of a modest amount of soy protein (25 g) produces a highly significant reduction of total cholesterol and LDL-C levels equivalent to ca. 6% LDL reduction [79]. Old studies were based on the effects in severely hypercholesterolaemic individuals, whereas patients with hypercholesterolaemia in the very highest range (>3350 mg/L) have not been selected for treatment in recent studies.

Soy products contain many isoflavonoids (genistein, daidzein, glycitin) that are natural phytoestrogens able to inhibit LDL oxidation, thus decreasing the risk of atherosclerosis [80]. Several studies have reported a decrease in susceptibility of LDL particles to oxidation with soy protein consumption [81]. Furthermore, soy protein rich in isoflavones reduced the susceptibility of LDL particles to oxidation in healthy subjects [82]. Meta-analyses of randomized controlled trials have shown that soy isoflavones can lower serum total and LDL cholesterol in humans [83]. The efficacy of soy foods and isoflavone supplements on blood lipids in clinical trials is less clear. These contradictory data may be due to poorer responses in hypercholesterolemic subjects compared to their control counterparts [74]. Recent clinical trials in postmenopausal women with soy protein have also shown similar discrepancies [84]. Clearly additional studies are needed to determine whether there are differences among whole food, soy protein and isoflavone extracts.

Comparisons between different animal or clinical studies are hampered by the lack of standardization of soy nomenclature, the different formulations, doses, routes of administration, time, and duration of exposure [85].

### 4.6. Dark Chocolate

Cocoa is a flavonoid-rich food that has been recently investigated for its possible role in the prevention of CVD [86, 87]. In healthy adults, drinking flavonoid-rich cocoa may improve NO-dependent vasorelaxation and flow-mediated dilation in the brachial arteries [88]. Administration of dark chocolate in essential hypertensives reduced ambulatory blood pressure and serum LDL-C levels whereas white chocolate had no effects [89]. Furthermore there was a clear reduction of the blood cholesterol levels as well as a significant rise of HDL cholesterol in addition to a marked reduction of circulating oxidized LDL [90].

### 4.7. Coffee and Tea

The active constituents of coffee apparently responsible for cardioprotective effect are diterpenes, such as kahweol and cafestol. Coffee consumption may possibly reduce the risk of myocardial infarction, but data are as yet inconclusive [91, 92]. A dose-response decrease in cardiovascular risk and heart disease mortality was reported for a daily caffeine intake in patients with type 2 diabetes [93, 94].

Green tea consumption appears to protect from CVD [95], but results are again inconsistent. It has been reported in a meta-analysis that the incidence of myocardial infarction among individuals who consumed three cups of tea daily was not statistically significant and there has been large variability across studies [96]. There were regional differences in this meta-analysis, with increasing tea consumption associated with an increased risk for CHD in the United Kingdom and for stroke in Australia, whereas the risk decreased in other regions, particularly in continental Europe. The hypothesis that addition of milk to tea (as typically done in United Kingdom and Australia) abolishes its plasma antioxidant potential may only partially explain these geographic differences.

## 5. Bioactive Dietary Compounds with Cardioprotective Potentials

Early research evaluated the benefits of plant-derived foods based on their vitamin C, vitamin E, and carotenoid content [24]. More recent work pointed out correlation of benefits with individual compounds [97]. However, the effects noted by testing them alone may be related to the synergistic action of the myriad of other bioactives present in source materials [98]. The main doubt about their efficacy, whether they should be consumed in a whole food diet or provided in a supplemental form remains to be answered and will be discussed in what follows. In each family of bioactive compounds there are usually many members that are present as discussed in following.

### 5.1. Phytochemicals

Plant foods contain many bioactive compounds known as “phytochemicals.” Some groups of phytochemicals which have or appear to have significant health potentials are carotenoids, phenolic compounds (flavonoids, phytoestrogens, phenolic acids), phytosterols and phytostanols, tocotrienols, organosulfur compounds, and nondigestible carbohydrates (dietary fiber and prebiotics). Isoflavones are found in high concentration in soybean, soybean products (e.g., tofu), and red clover. Lignans are mainly found in flaxseed.

#### 5.1.1. Polyphenol Compounds

Polyphenols have been shown in *in vivo* studies to exert antiatherosclerotic effects in the early stages of atherosclerosis development (e.g., decrease LDL oxidation); improve endothelial function and increase nitric oxide release (potent vasodilator); modulate inflammation and lipid metabolism (i.e., hypolipidemic effect); improve antioxidant status; protect against atherothrombotic episodes including myocardial ischemia and platelet aggregation (Perez-Jimenez and Saura-Calixto, 2008) [99].

#### 5.1.2. Flavonoids

Plant-derived flavonoids are the most common group of polyphenols in the human diet, and are contained in vegetables and fruits as well as in beverages such as cocoa, tea, and wine. Some isoflavones like lignans are phytoestrogens, a group of nonsteroidal plant constituents that elicit estrogen-like biological response. They are associated as minor components with dietary fiber in dietary items like oilseeds, cereal grains, vegetables, fruits, and legumes. Like other phenolic compounds, phytoestrogens have antioxidant activity, and like estrogens, they can influence lipoprotein metabolism and enhance vascular reactivity.

Intake of flavonoids has been associated with decreased cardiovascular mortality and general mortality among elderly Dutch individuals [100]. Several prospective studies have reported inverse associations between flavonoid intake and CVD incidence or mortality [101–103]. Within the cardiovascular protective mechanisms of flavonoids, several mechanisms have been proposed to explain the anti-inflammatory properties of flavonoids. These include their antioxidant activity and their properties as metal chelators, for transitional elements such as copper and iron that catalyze lipid oxidation; inhibitors of platelet aggregation; modulation of the activity of eicosanoid generating enzymes in inflammatory cells enhancers of nitric oxide synthesis; lowering of superoxide production; beneficial effects on lipid profile [104, 105] and modulation of proinflammatory gene expression [97, 106, 107].

A systematic review of the effectiveness of different flavonoid subclasses and flavonoid-rich foods on CVD concluded that some flavonoid-rich foods, including chocolate or cocoa, red wine or grape, and green or black tea may have some measurable effects on CVD risk factors, including a reduction in blood pressure and a favorable influence on endothelial function [108]. Nevertheless, there still exists uncertainty as to whether or not flavonoids are the only bioactive compounds mediating the enhanced vascular reactivity. This is in part due to the fact that flavonoid-rich food and plant extracts contain many potentially bioactive compounds, and information ensuing from investigations in humans using specific, chemically pure flavonoids is rare. Therefore, it cannot be completely excluded, that the observed effects on vascular function may potentially, at least in part, be related to compounds other than flavonoids contained in these foods/extracts. It should be noted that no conclusive evidence has so far emerged about the therapeutic efficacy of isoflavones in reducing the incidence of CVD and breast cancer and in preventing the loss of bone mineral density in menopausal women.

#### 5.1.3. Plant Sterols and Stanols

Plant sterols or phytosterols are structurally similar and functionally analogous to the animal sterol, cholesterol. A less abundant class of related compounds is the plant stanols or phytostanols, which are completely saturated forms of phytosterols. Dietary sources include vegetable oils, nuts, seed and grains, but the amounts are often not large enough to have significant cholesterol-lowering effects [109]. Also they have been incorporated in foods with higher fat content, such as spreads (margarines) and salad dressings. Phytosterols and phytostanols inhibit intestinal absorption of cholesterol [110]. HDL and/or VLDL were generally not affected by stanols/sterols intake. Yet, effects of sterols/stanols on LDLs have been found to be additive to diets and/or cholesterol-lowering drugs [111]. This has been the basis for the development of phytosterol-enriched functional foods. Similar efficacy has been observed between plant sterols and stanols when they are esterified, which is the form added to foods [112]. Because plant sterols and stanols can reduce fat-soluble vitamins, it is necessary to consume plant sterols and stanols with an appropriate intake of fruit and vegetables, including carotenoids [113]. There are also concerns about margarines containing plant sterols and stanols, related to the energy intake associated with consuming >2 g daily [114].

#### 5.1.4. Vitamin C

The powerful antioxidant functions of vitamin C serve to reduce tissue reactive oxygen species concentrations, which in the atherosclerotic condition help prevent endothelial dysfunction, inhibit vascular smooth muscle proliferation, and reduce oxidized LDL cholesterol [115]. Several prospective studies have assessed the role of vitamin C, both dietary and supplemental, in CVD, with mixed results [116]. Despite its role as an antioxidant, vitamin C has been identified as a pro-oxidant under conditions of high oxidative stress [117]. Most clinical trials have incorporated vitamin C into a mixture including vitamin E and *β*-carotene, with largely null results in relation to CVD [118, 119]. Nutrients and bioactive compounds in foods act synergistically or antagonistically in the complex food matrix to deliver the established health effects of foods.

#### 5.1.5. Carotenoids

Carotenoids have been credited with other health-promoting effects: immune enhancement and reduction of the risk of developing degenerative diseases such as cancer, CVD, and cataract [120, 121]. These physiological activities have been attributed to an antioxidant property, specifically to the ability to quench singlet oxygen and interact with free radicals [122]. The carotenoids, particularly lycopene and beta-carotene, are other dietary antioxidants that function to reduce oxidative stress *in vivo* and blood markers of inflammation [123]. Evidence for a role of carotenoids in CVD first stemmed from studies that showed that higher intakes of fruit and vegetables were associated with lower risk of CVD [124]. However, studies on the association between dietary carotenoids and CVD risk have been inconsistent. Women who regularly eat large amounts of lycopene, from tomato and derivatives, are less prone to developing CVD, since this phytochemical has the strongest antioxidant activities in the cardiovascular system [125]. Results from the KIHD study confirmed that lower serum lycopene levels were associated with enhanced risk of atherosclerosis in the common carotid artery [58]. These protective associations were not evident for other carotenoids like lutein, zeaxanthin, or *β*-cryptoxanthin [126]. Despite overwhelming evidence from epidemiological studies on carotenoids role in CVD, clinical trials have failed to demonstrate a beneficial effect [18, 127].

#### 5.1.6. Vitamin E

In addition to its role as a free radical scavenger, vitamin E is a potent anti-inflammatory agent, especially at high doses [128]. Mounting evidence supports the strong inverse association between plasma vitamin E and CVD [129] as well as that between vitamin E intake and risk of CHD [130]. Nevertheless, clinical trials failed to support the role of vitamin E supplementation in preventing CVD [131]. Subsequent meta-analyses and systematic reviews of more than 90 trials showed similar null results [132, 133]. Most recently, a dose-response meta-analysis showed increased risk of high-dose vitamin E (≥400 IU/day) on total mortality [17]. There are many potential explanations for these largely negative effects that include the use of the most appropriate form and/or dose of vitamin E. This may be essential to obtain effective reduction of oxidative stress. As with carotenoids, the contrast between observational and interventional studies results suggests that the protective effects of *α*-tocopherol occur in the presence of other nutrients, and, therefore, it is most effective and safe when obtained from foods.

### 5.2. Antioxidant Vitamin Supplementation

The term “dietary supplement” can be defined as a product that is intended to supplement the diet with one or more of the following dietary ingredients: a vitamin, a mineral, a herb or other botanical, an amino acid, intended for ingestion in pill, capsule, tablet, or liquid form [134]. It should be noted that nutraceuticals differ from dietary supplements in the following aspects: (1) nutraceuticals must not only supplement the diet but should also aid in the prevention and/or treatment of disease, and (2) nutraceuticals are used as conventional foods or as sole items of a meal or diet [134].

In view of the detrimental role of free radicals and reactive oxygen species in the pathophysiology of atherosclerosis, supplementation with antioxidants (vitamins A, C, and E, folic acid, *β*-carotene, selenium, and zinc) was expected to be protective. Some supplements (e.g., marine n-3 FAs and niacin) are effective in improving CVD risk factors, whereas others (like B-vitamins: folate, vitamin B12, vitamin B6, antioxidants; vitamin E and selenium) despite promising *in vitro* studies have shown little effect on CVD mortality and morbidity, and antioxidant supplements may even have adverse effects.

Epidemiologic studies have reported that a high dietary intake of foods rich in vitamin E [21], vitamin C [135], and *β*-carotene [126] have been inversely associated with the incidence of CAD. Nevertheless, firm recommendations to take antioxidant supplements to treat or prevent CVD or metabolic diseases require evidence derived from randomized controlled trials with vitamin supplements, which were found to be disappointing [136]. Indeed, only one trial has shown a reduction in myocardial infarction and cardiac events [137], whereas all the others have shown no effect or detrimental effects. Within five trials, such antioxidant supplementation was associated with increased all-cause mortality and two have shown higher risk of fatal CHD (ATBC and CARET). Indeed, those controversial results by no means invalidate the role of oxidative stress in CVD; rather, they suggest that very high supplementation with antioxidant vitamins may not represent an optimal strategy to prevent vascular damage induced by oxidative stress and lipid oxidation. Several factors ought to be considered, which may have contributed to cloud the results of clinical trials, like choosing the optimal dose and form of vitamins, the use of single vitamin(s) or in combination, and so forth [138].

## 6. Dietary Patterns and Reduced Risk of Chronic Diseases

Eating habits and dietary trends have health, environmental, and social impacts. Some diet plans demonstrated the ability to reduce cardiovascular risk [139]. Despite the high levels of interest in the diet and health relationship, the traditional approach in nutritional epidemiology has mainly focused on the effects of individual nutrients or foods. However, individuals do not consume nutrients in isolation but, rather, meals consisting of a variety of foods in combinations of nutrients that are likely to be interactive or synergistic. Indeed much less concern has been focused on dietary patterns because of their complex nature. The mechanisms by which these diets reduce inflammatory risk are not well understood but may relate to high intakes of food items containing antioxidant nutrients and polyphenols that reduce free radicals concentrations throughout tissues.

Consuming patterns of food vary significantly across nations, and this might contribute differently to the apparent differences in the health of populations on the continent.

### 6.1. Mediterranean Diet

The Mediterranean diets contain high levels of fruits, vegetables, cereals, beans, nuts and seeds, and olive oil, with little red meat and dairy products. Fish and poultry are consumed in low-to-moderate amounts; and wine is consumed in moderation. They have received great attention and numerous reports have demonstrated low rates of chronic disease among populations known to consume these diets [140]. Moreover, clinical trials have confirmed their cardiovascular protective effects and their effectiveness to reduce inflammatory markers in high-risk populations [13, 141, 142]. Several Mediterranean diet foods, including polyunsaturated fat products, vegetables, fruit, whole grains, legumes, and low glycaemic index starchy foods with functional properties may protect against type 2 diabetes [143]. They were also shown to reduce serum homocysteine concentrations and consequently the risk of coronary events, especially in high-risk individuals [144]. Olive oil (which induces a high ratio of monounsaturated to saturated lipids) appears to be chiefly responsible for the apparent protection offered by the Mediterranean diet against hypertension [145]. The high antioxidant content of plant foods and olive oil may also contribute to the health of the vascular system. However, it has been indicated in a recent review that not all components of the Mediterranean diet are equally protective [146]. Therefore, the convincing evidence for the protective role of the Mediterranean diet provided by the Lyon Diet Heart Study [141] needs to be replicated in primary prevention trials as well as the conduction in non-Mediterranean populations to determine if the favorable effects transfer to other groups.

### 6.2. DASH Diet

The Dietary Approaches to Stop Hypertension (DASH) trial reported that a diet rich in fish, fruit, vegetables, whole grains, and nuts and low in fat dairy foods significantly lowered systolic blood pressure in patients with isolated systolic hypertension [147]. This dietary pattern also limits saturated fat, red meat, sweets, and sugar-containing beverages. Consistent weight loss induced by a very low caloric diet improves vasodilatation-mediated blood flow, an effect associated with decreased glucose blood levels [148]. Regular physical exercise, decreased salt (NaCl) intake, moderate alcoholic ingestion, and an increase in dietary potassium constitute evidence-based strategies to reduce blood pressure by DASH diet.

Because hypertension is a CVD risk factor, several prospective cohort studies have examined associations between adherence to a DASH dietary pattern and incident CVD events [10, 149, 150]. In addition to its effects on blood pressure and incident CHD, the DASH diet appears to have beneficial effects on several CVD risk factors, including total cholesterol, LDL-C [151], inflammation [149], and homocysteine [152]. As a whole, the evidence for the protective role of the DASH dietary pattern in prevention of CVD is strong.

### 6.3. Portfolio Diet

Step I and Step II diets are early examples of dietary strategies recommended for the clinical management of high blood cholesterol [153]. The Step I diet requires a total fat intake of less than 30% of total calories, with saturated fatty acids contributing to less than 10% of total calories and cholesterol less than 300 mg/day. For individuals who require a more aggressive approach to meet their LDL-C goals, the Step II diet, which lowers saturated fatty acids to less than 7% of total calories and cholesterol to less than 200 mg/day, is recommended.

Current recommendations for CVD risk reduction continue to focus on LDL-C as a major therapeutic target. The National Cholesterol Education Program (NCEP) Adult Treatment Panel (ATP) III guidelines recommend therapeutic lifestyle changes for individuals whose LDL-C levels are above goal (2001). LDL-C lowering can also be achieved by adding other dietary components, specifically plant sterols, viscous fibers, soy protein, and almonds. This combination of dietary components has been labeled the Portfolio diet [154]. Another important attribute of the Portfolio diet is its beneficial effects on C-reactive protein lowering, a strong independent predictor of cardiovascular risk [155].

### 6.4. Vegetarian Diet

A vegetarian diet, devoid of meat and fish, and high in fruits, vegetables, and nuts, rich sources of the antioxidant nutrients and polyphenols, contribute to the anti-inflammatory potential of these diets. In terms of micronutrients content, vegan diets are usually higher in magnesium, folic acid, vitamins C and E, iron, and phytochemicals, and they tend to be lower in n-3 fatty acids, vitamin D, calcium, zinc, and vitamin B-12. Much of this benefit is likely related to the low body weights, low blood pressure, and low blood cholesterol concentrations generally observed for vegetarians due to their lower intakes of saturated fats, cholesterol, and calories. Vegetarians are reported to have a lower risk of dying from ischemic heart disease [156] and have a reduced all-cause mortality [57].

### 6.5. Okinawan Diet

Equally notable is the wide variation in other aspects of healthy diets such as macronutrient intake, represented most notably by the healthy Okinawan diet, which is low in fat and high in carbohydrates (mostly from vegetable sources). This suggests that low-energy, nutrient-dense diets with high-quality carbohydrates may be beneficial for reducing the risk of CVD among many chronic diseases [157]. The cardioprotective benefit of Okinawan diet is ascribed, in part, to the low consumption of saturated fat. Other possible mechanisms, such as the high contents of phytochemicals, high antioxidant intake, and low glycemic load in this diet, are also likely to be contributing to decreased risk for CVD and some cancers through multiple mechanisms, including reduced oxidative stress. A comparison of the nutrient profiles of the previous dietary patterns shows that the traditional Okinawan diet is the lowest in fat intake, particularly in terms of saturated fat, and highest in carbohydrate intake, in keeping with the very high intake of antioxidant-rich yet calorie-poor orange-yellow root vegetables, such as sweet potatoes, and green leafy vegetables [158]. The longevity of Okinawan populations suggests that such a diet may even help to slow the aging process itself [159].

## 7. Conclusion

The relationship between dietary factors and CVD has been a major focus of health research for almost half a century. Epidemiological and clinical studies indicate that the risk of CVD is reduced by a diet rich in fruits, vegetables, unrefined grains, fish and low-fat dairy products, and low in saturated fats and sodium [11]. Other foods such as mono- and polyunsaturated fats, brans, nuts, plant sterols, and soy proteins have all been shown to have a favorable effect on lipid profile and blood pressure [9, 161].

Novel dietary approaches to cardiovascular prevention are of major significance in clinical research and practice. However, nutrition is a very complex research topic and it is not clear whether an individual component of the diet or a combination of nutrients and dietary habits may be responsible for any cardioprotective effects. The advances in the knowledge of both the disease processes and healthy dietary components have provided new avenues to develop dietary strategies to prevent and/or to treat CVD. It is now evident, based on the extensive scientific evidence, that functional foods have broad ranging physiologic effects *in vivo* that lessen inflammatory cascades and vascular reactivity. These effects are as powerful as pharmaceutical interventions, yet much safer. Although many of these functional foods have been found to have high therapeutic potential, future studies should include well-designed clinical trials assessing different combinations of these nutrients to realize possible additive and/or synergistic effects on health outcomes. Many functional foods have antioxidant and anti-inflammatory activities, by mechanisms that may require further investigation. Therefore, these functional foods should be incorporated into a healthy diet to provide cardiovascular benefits and hence lower cardiovascular risk.

Emerging evidence for a potential role of antioxidant vitamins in atherosclerotic progression implies that the effect of micronutrients is complex and not likely due to a single nutrient in isolation. Therefore, the use of vitamin supplements is not recommended. Rather, efforts should be targeted to increasing the consumption of vitamins-rich fruit and vegetables.

## Figures and Tables

**Table 1:** Cardiovascular risk factors.

Category	Examples
Nonmodifiable risk factors	Advancing age
	Male gender
	Family history/genotype

Metabolic risk factors	Hypertension
	Hyperlipidemia
	Diabetes mellitus
	Metabolic syndrome
	Obesity/overweight

Lifestyle risk factors	Diet
	Smoking
	Physical activity

Novel risk factors	Elevated homocysteine level
	Elevated lipoprotein (a) level
	Small dense LDL-C
	Elevated inflammatory markers levels
	Elevated hemostatic factors levels

**Table 2:** Potential cardiovascular protective effects of functional foods.

Functional foods	Bioactive compounds	Potential mechanism	References
- Nuts	- Tocopherols, omega-3 fatty acids	Lowering blood cholesterol	Sabate and Ang [60] Albert et al. [62]
- Legumes	- Fiber and polyphenols		Erkkilä and Lichtenstein [55]
- Fruits and vegetables	- Fiber (pectin)		Liu et al. [56]
- Margarine	- Phytosterols		Darmadi-Blackberry et al. [63]
- Fish oil	- Omega-3 fatty acids		Hicks and Moreau [110]
- Whole grains	- Fiber and phytochemicals		Dyerberg et al. [37]
- Soy proteins	- Genistein and daidzein		Lutsey et al. [73]
- Dark chocolate	- Flavonoid		Anderson et al. [78] Harland and Haffner [79] Grassi et al. [89] Baba et al. [90]

- Fish	- Omega-3 fatty acids	Inhibition of LDL-C oxidation	Lee and Wander [39]
- Green leafy vegetables, fruits	- Carotenoids		Chopra et al. [52]
- Citrus fruits and vegetables	- Vitamin C		Sesso et al. [119]
- Tomato	- Lycopene		Aguirre and May [115]
- Extravirgin olive oil	- Polyphenolics and oleic acid		Engelhard et al. [123]
- Green tea	- Tea polyphenolics		Psaltopoulou et al. [145]
- Soy proteins	- Genistein, daidzein, and glycitein		Wiseman et al. [81]
- Dark chocolate	- Flavonoid		Grassi et al. [89]
- Pomegranate	- Polyphenols		Davidson et al. [99]

- Fish	- Omega-3 fatty acids	Lowering blood triglycerides	Durrington et al. [36] Dyerberg et al. [37] Harris et al. [40]

- Fish	- Omega-3 fatty acids		Mozaffarian [35]
- Legumes	- Fiber		He et al. [53]
- Whole grains	- Fiber and phytochemicals		Savica et al. [54] Lee et al. [65]
- Citrus fruits	- Ascorbic acid		Flint et al. [72]
		Decreasing blood pressure	
- Ginseng	- Ginsenosides		Hooper et al. [108]
- Onion and garlic	- Quercetin		
- Green and black teas	- Tea polyphenols		
- Grapes and red wines	- Grape polyphenols		Perez-Jimenez and Saura-Calixto [160]
- Dark chocolate	- Flavonoid		Grassi et al. [89]

- Fruits and vegetables	- Folate - Phytochemicals	Lowering blood homocysteine	Samman et al. [144] Appel et al. [152]
- Whole grains	- Fiber and phytochemicals		Jenkins et al. [154]
- Citrus fruits and vegetables	- Vitamin C		Broekmans et al. [27]
- Nuts, seeds, and oils	- Vitamin E		

- Tomatoes	- Lycopene	Antioxidant action	Engelhard et al. [123] Krinsky and Johnson [121] Sesso et al. [119] Aguirre and May [115]
- Green leafy vegetables, fruits	- Carotenoids		
- Vegetable oils	- Tocopherol, tocotrienols		
- Citrus fruits and vegetables	- Vitamin C		Mink et al. [105] Perez-Jimenez and Saura-Calixto [160]
- Soy proteins	- Genistein and daidzein		
- Green and black teas	- Tea polyphenols		
- Grapes and red wines	- Anthocyanins, catechins, cyanidins, and flavonols, myricetin and quercetin		

- Nuts, seeds, and oils	- Vitamin E	Anti-inflammatory action	Singh et al. [128] Kushi et al. [130]
- Fish	- Omega-3 fatty acids		Ueeda et al. [38]
- Legumes	- Polyphenols		Lutsey et al. [73]
- Grapes and red wines	- Anthocyanins, catechins, cyanidins, and flavonols, myricetin, and quercetin		Perez-Jimenez and Saura-Calixto [160]

- Fish	- Omega-3 fatty acids	Endothelial function	Ueeda et al. [38]
- Nuts	- Polyphenols		Ros et al. [61]
- Citrus fruits and vegetables	- Vitamin C		Aguirre and May [115]
- Grapes and red wines	- Anthocyanins, catechins, cyanidins, and flavonols, myricetin, and quercetin		Perez-Jimenez and Saura-Calixto [160]
- Dark chocolate	- Flavonoid		Grassi et al. [88]

- Grapes and red wines	- Anthocyanins, catechins, cyanidins, and flavonols, myricetin and quercetin	Platelets aggregation	Perez-Jimenez and Saura-Calixto [160] Mink et al. [105]

## References

[1] De Backer G, Ambrosioni E, Borch-Johnsen K (2003). European guidelines on cardiovascular disease prevention in clinical practice. *Atherosclerosis*.

[2] Alberti KGMM, Eckel RH, Grundy SM (2009). Harmonizing the metabolic syndrome: a joint interim statement of the international diabetes federation task force on epidemiology and prevention; National heart, lung, and blood institute; American heart association; World heart federation; International atherosclerosis society; and international association for the study of obesity. *Circulation*.

[3] McGill HC (1979). The relationship of dietary cholesterol to serum cholesterol concentration and to atherosclerosis in man. *American Journal of Clinical Nutrition*.

[4] He K, Merchant A, Rimm EB (2003). Dietary fat intake and risk of stroke in male US healthcare professionals: 14 year prospective cohort study. *British Medical Journal*.

[5] Bernstein AM, Sun Q, Hu FB, Stampfer MJ, Manson JE, Willett WC (2010). Major dietary protein sources and risk of coronary heart disease in women. *Circulation*.

[6] Bjelakovic G, Gluud C (2007). Surviving antioxidant supplements. *Journal of the National Cancer Institute*.

[7] Ross S (2000). Functional foods: the food and drug administration perspective. *American Journal of Clinical Nutrition*.

[8] Stampfer MJ, Hu FB, Manson JE, Rimm EB, Willett WC (2000). Primary prevention of coronary heart disease in women through diet and lifestyle. *New England Journal of Medicine*.

[9] Reddy KS, Katan MB (2004). Diet, nutrition and the prevention of hypertension and cardiovascular diseases. *Public Health Nutrition*.

[10] Folsom AR, Parker ED, Harnack LJ (2007). Degree of concordance with DASH diet guidelines and incidence of hypertension and fatal cardiovascular disease. *American Journal of Hypertension*.

[11] Aldana SG, Greenlaw R, Salberg A, Merrill RM, Hager R, Jorgensen RB (2007). The effects of an intensive lifestyle modification program on carotid artery intima-media thickness: a randomized trial. *American Journal of Health Promotion*.

[12] Kris-Etherton PM, Lichtenstein AH, Howard BV, Steinberg D (2004). Antioxidant vitamin supplements and cardiovascular disease. *Circulation*.

[13] Huang CL, Sumpio BE (2008). Olive oil, the Mediterranean diet, and cardiovascular health. *Journal of the American College of Surgeons*.

[14] Ferrari CKB, Torres EAFS (2003). Biochemical pharmacology of functional foods and prevention of chronic diseases of aging. *Biomedicine and Pharmacotherapy*.

[15] Tiana L, Caib Q, Wei H (1998). Alterations of antioxidant enzymes and oxidative damage to macromolecules in different organs of rats during aging. *Free Radical Biology and Medicine*.

[16] Forster MJ, Sohal BH, Sohal RS (2000). Reversible effects of long-term caloric restriction protein oxidative damage. *Journals of Gerontology Series A*.

[17] Miller ER, Pastor-Barriuso R, Dalal D, Riemersma RA, Appel LJ, Guallar E (2005). Meta-analysis: high-dosage vitamin E supplementation may increase all-cause mortality. *Annals of Internal Medicine*.

[18] Bjelakovic G, Nikolova D, Gluud LL, Simonetti RG, Gluud C (2007). Mortality in randomized trials of antioxidant supplements for primary and secondary prevention: systematic review and meta-analysis. *Journal of the American Medical Association*.

[19] Ferrari CKB (2004). Functional foods, herbs and nutraceuticals: towards biochemical mechanisms of healthy aging. *Biogerontology*.

[20] DeFelice SL (1995). The nutraceutical revolution: its impact on food industry R&D. *Trends in Food Science and Technology*.

[21] Rimm EB, Stampfer MJ, Ascherio A, Giovannucci E, Colditz GA, Willett WC (1993). Vitamin E consumption and the risk of coronary heart disease in men. *New England Journal of Medicine*.

[22] Stampfer MJ, Hennekens CH, Manson JE, Colditz GA, Rosner B, Willett WC (1993). Vitamin E consumption and the risk of coronary disease in women. *New England Journal of Medicine*.

[23] Thomas PR, Earl R (1994). Enhancing the food supply. *Opportunities in the Nutrition and Food Sciences*.

[24] Block G, Patterson B, Subar A (1992). Fruit, vegetables, and cancer prevention: a review of the epidemiological evidence. *Nutrition and Cancer*.

[25] Mattson MP (2003). Will caloric restriction and folate protect against AD and PD?. *Neurology*.

[26] Smolders RGV, Sipkema P, Kenemans P, Stehouwer CDA, Van Der Mooren MJ (2004). Homocysteine impairs estrogen-induced vasodilation in isolated rat arterioles. *Menopause*.

[27] Broekmans WMR, Klöpping-Ketelaars IAA, Schuurman CRWC (2000). Fruits and vegetables increase plasma carotenoids and vitamins and decrease homocysteine in humans. *Journal of Nutrition*.

[28] McKeown NM, Meigs JB, Liu S, Wilson PWF, Jacques PF (2002). Whole-grain intake is favorably associated with metabolic risk factors for type 2 diabetes and cardiovascular disease in the Framingham Offspring Study. *American Journal of Clinical Nutrition*.

[29] Block G, Mangels AR, Norkus EP, Patterson BH, Levander OA, Taylor PR (2001). Ascorbic acid status and subsequent diastolic and systolic blood pressure. *Hypertension*.

[30] Kromhout D, Feskens EJM, Bowles CH (1995). The protective effect of a small amount of fish on coronary heart disease mortality in an elderly population. *International Journal of Epidemiology*.

[31] He K, Song Y, Daviglus ML (2004). Accumulated evidence on fish consumption and coronary heart disease mortality: a meta-analysis of cohort studies. *Circulation*.

[32] Bjerregaard LJ, Joensen AM, Dethlefsen C (2010). Fish intake and acute coronary syndrome. *European Heart Journal*.

[33] Carrero JJ, Baró L, Fonollá J (2004). Cardiovascular effects of milk enriched with *ω*-3 polyunsaturated fatty acids, oleic acid, folic acid, and vitamins e and B6 in volunteers with mild hyperlipidemia. *Nutrition*.

[34] Mozaffarian D, Rimm EB (2006). Fish intake, contaminants, and human health evaluating the risks and the benefits. *Journal of the American Medical Association*.

[35] Mozaffarian D (2008). Fish and n-3 fatty acids for the prevention of fatal coronary heart disease and sudden cardiac death. *American Journal of Clinical Nutrition*.

[36] Durrington PN, Bhatnagar D, Mackness MI (2001). An omega-3 polyunsaturated fatty acid concentrate administered for one year decreased triglycerides in simvastatin treated patients with coronary heart disease and persisting hypertriglyceridaemia. *Heart*.

[37] Dyerberg J, Eskesen DC, Andersen PW (2004). Effects of trans- and n-3 unsaturated fatty acids on cardiovascular risk markers in healthy males. An 8 weeks dietary intervention study. *European Journal of Clinical Nutrition*.

[38] Ueeda M, Doumei T, Takaya Y (2008). Serum N-3 polyunsaturated fatty acid levels correlate with the extent of coronary plaques and calcifications in patients with acute myocardial infarction. *Circulation Journal*.

[39] Lee YS, Wander RC (2005). Reduced effect on apoptosis of 4-hydroxyhexenal and oxidized LDL enriched with n-3 fatty acids from postmenopausal women. *Journal of Nutritional Biochemistry*.

[40] Harris WS, Miller M, Tighe AP, Davidson MH, Schaefer EJ (2008). Omega-3 fatty acids and coronary heart disease risk: clinical and mechanistic perspectives. *Atherosclerosis*.

[41] Hu FB, Stampfer MJ, Manson JE (1999). Dietary intake of *α*-linolenic acid and risk of fatal ischemic heart disease among women. *American Journal of Clinical Nutrition*.

[42] Campos H, Baylin A, Willett WC (2008). *α*-Linolenic acid and risk of nonfatal acute myocardial infarction. *Circulation*.

[43] Djoussé L, Arnett DK, Carr JJ (2005). Dietary linolenic acid is inversely associated with calcified atherosclerotic plaque in the coronary arteries: the National Heart, Lung, and Blood Institute Family Heart Study. *Circulation*.

[44] Kris-Etherton PM, Harris WS (2002). Fish consumption, fish oil, omega-3 fatty acids, and cardiovascular disease. *Circulation*.

[45] Liu S, Manson JE, Lee IM (2000). Fruit and vegetable intake and risk of cardiovascular disease: the Women’s Health Study. *American Journal of Clinical Nutrition*.

[46] Joshipura KJ, Hu FB, Manson JE (2001). The effect of fruit and vegetable intake on risk for coronary heart disease. *Annals of Internal Medicine*.

[47] Dauchet L, Amouyel P, Hercberg S, Dallongeville J (2006). Fruit and vegetable consumption and risk of coronary heart disease: a meta-analysis of cohort studies. *Journal of Nutrition*.

[48] He FJ, Nowson CA, Lucas M, MacGregor GA (2007). Increased consumption of fruit and vegetables is related to a reduced risk of coronary heart disease: meta-analysis of cohort studies. *Journal of Human Hypertension*.

[49] Lock K, Pomerleau J, Causer L, Altmann DR, McKee M (2005). The global burden of disease attributable to low consumption of fruit and vegetables: implications for the global strategy on diet. *Bulletin of the World Health Organization*.

[50] Pietinen P, Rimm EB, Korhonen P (1996). Intake of dietary fiber and risk of coronary heart disease in a cohort of Finnish men: the Alpha-Tocopherol, Beta-Carotene Cancer Prevention Study. *Circulation*.

[51] Rosengren A, Subramanian SV, Islam S (2009). Education and risk for acute myocardial infarction in 52 high, middle and low-income countries: INTERHEART case-control study. *Heart*.

[52] Chopra M, O’Neill ME, Keogh N, Wortley G, Southon S, Thurnham DI (2000). Influence of increased fruit and vegetable intake on plasma and lipoprotein carotenoids and LDL oxidation in smokers and nonsmokers. *Clinical Chemistry*.

[53] He J, Klag MJ, Whelton PK, Chen JY, Qian MC, He GQ (1995). Dietary macronutrients and blood pressure in southwestern China. *Journal of Hypertension*.

[54] Savica V, Bellinghieri G, Kopple JD (2010). The the effect of nutrition on blood pressure. *Annual Review of Nutrition*.

[55] Erkkilä AT, Lichtenstein AH (2006). Fiber and cardiovascular disease risk: how strong is the evidence?. *Journal of Cardiovascular Nursing*.

[56] Liu S, Buring JE, Sesso HD, Rimm EB, Willett WC, Manson JE (2002). A prospective study of dietary fiber intake and risk of cardiovascular disease among women. *Journal of the American College of Cardiology*.

[57] Genkinger JM, Platz EA, Hoffman SC, Comstock GW, Helzlsouer KJ (2004). Fruit, vegetable, and antioxidant intake and all-cause, cancer, and cardiovascular disease mortality in a community-dwelling population in Washington County, Maryland. *American Journal of Epidemiology*.

[58] Rissanen TH, Voutilainen S, Nyyssönen K, Salonen R, Kaplan GA, Salonen JT (2003). Serum lycopene concentrations and carotid atherosclerosis: the Kuopio Ischaemic Heart Disease Risk Factor Study. *American Journal of Clinical Nutrition*.

[59] Fraser GE, Sabate J, Beeson WL, Strahan TM (1992). A possible protective effect of nut consumption on risk of coronary heart disease: the adventist health study. *Archives of Internal Medicine*.

[60] Sabaté J, Ang Y (2009). Nuts and health outcomes: new epidemiologic evidence. *American Journal of Clinical Nutrition*.

[61] Ros E, Núñez I, Pérez-Heras A (2004). A walnut diet improves endothelial function in hypercholesterolemic subjects: a randomized crossover trial. *Circulation*.

[62] Albert CM, Michael Gaziano J, Willett WC, Manson JE (2002). Nut consumption and decreased risk of sudden cardiac death in the physicians’ health study. *Archives of Internal Medicine*.

[63] Darmadi-Blackberry I, Wahlqvist ML, Kouris-Blazos A (2004). Legumes: the most important dietary predictor of survival in older people of different ethnicities. *Asia Pacific Journal of Clinical Nutrition*.

[64] Martins JM, Riottot M, De Abreu MC (2005). Cholesterol-lowering effects of dietary blue lupin (Lupinus angustifolius L.) in intact and ileorectal anastomosed pigs. *Journal of Lipid Research*.

[65] Lee YP, Mori TA, Puddey IB (2009). Effects of lupin kernel flour-enriched bread on blood pressure: a controlled intervention study. *American Journal of Clinical Nutrition*.

[66] Morris JN, Marr JW, Clayton DG (1977). Diet and heart: a postscript. *British Medical Journal*.

[67] Jacobs DR, Pereira MA, Meyer KA, Kushi LH (2000). Fiber from whole grains, but not refined grains, is inversely associated with all-cause mortality in older women: the Iowa Women’s Health Study. *Journal of the American College of Nutrition*.

[68] Bazzano LA, Serdula MK, Liu S (2003). Dietary intake of fruits and vegetables and risk of cardiovascular disease. *Current Atherosclerosis Reports*.

[69] Lupton JR, Turner ND (2003). Dietary fiber and coronary disease: does the evidence support an association?. *Current Atherosclerosis Reports*.

[70] Streppel MT, Arends LR, Van’t Veer P, Grobbee DE, Geleijnse JM (2005). Dietary fiber and blood pressure: a meta-analysis of randomized placebo-controlled trials. *Archives of Internal Medicine*.

[71] Mellen PB, Walsh TF, Herrington DM (2008). Whole grain intake and cardiovascular disease: a meta-analysis. *Nutrition, Metabolism and Cardiovascular Diseases*.

[72] Flint AJ, Hu FB, Glynn RJ (2009). Whole grains and incident hypertension in men. *American Journal of Clinical Nutrition*.

[73] Lutsey PL, Jacobs DR, Kori S (2007). Whole grain intake and its cross-sectional association with obesity, insulin resistance, inflammation, diabetes and subclinical CVD: the MESA Study. *British Journal of Nutrition*.

[74] Sacks FM, Lichtenstein A, Van Horn L, Harris W, Kris-Etherton P, Winston M (2006). Soy protein, isoflavones, and cardiovascular health: An American Heart Association Science Advisory for professionals from the Nutrition Committee. *Circulation*.

[75] Burslem J, Schonfeld G, Howald MA (1978). Plasma apoprotein and lipoprotein lipid levels in vegetarians. *Metabolism*.

[76] Zhang X, Shu XO, Gao YT (2003). Soy food consumption is associated with lower risk of coronary heart disease in Chinese women. *Journal of Nutrition*.

[77] Nagata C, Takatsuka N, Kurisu Y, Shimizu H (1998). Decreased serum total cholesterol concentration is associated with high intake of soy products in Japanese men and women. *Journal of Nutrition*.

[78] Anderson JW, Johnstone BM, Cook-Newell ME (1995). Meta-analysis of the effects of soy protein intake on serum lipids. *New England Journal of Medicine*.

[79] Harland JI, Haffner TA (2008). Systematic review, meta-analysis and regression of randomised controlled trials reporting an association between an intake of circa 25 g soya protein per day and blood cholesterol. *Atherosclerosis*.

[80] Wiseman H (1999). The bioavailability of non-nutrient plant factors: dietary flavonoids and phyto-oestrogens. *Proceedings of the Nutrition Society*.

[81] Wiseman H, O’Reilly JD, Adlercreutz H (2000). Isoflavone phytoestrogens consumed in soy decrease F2-isoprostane concentrations and increase resistance of low-density lipoprotein to oxidation in humans. *American Journal of Clinical Nutrition*.

[82] Jenkins DJA, Kendall CWC, Jackson CJC (2002). Effects of high- and low-isoflavone soyfoods on blood lipids, oxidized LDL, homocysteine, and blood pressure in hyperlipidemic men and women. *American Journal of Clinical Nutrition*.

[83] Taku K, Umegaki K, Sato Y, Taki Y, Endoh K, Watanabe S (2007). Soy isoflavones lower serum total and LDL cholesterol in humans: a meta-analysis of 11 randomized controlled trials. *American Journal of Clinical Nutrition*.

[84] Ho SC, Chen YM, Ho SSS, Woo JLF (2007). Soy isoflavone supplementation and fasting serum glucose and lipid profile among postmenopausal Chinese women: a double-blind, randomized, placebo-controlled trial. *Menopause*.

[85] Cederroth CR, Nef S (2009). Soy, phytoestrogens and metabolism: a review. *Molecular and Cellular Endocrinology*.

[86] Ding EL, Hutfless SM, Ding X, Girotra S (2006). Chocolate and prevention of cardiovascular disease: a systematic review. *Nutrition and Metabolism*.

[87] Galleano M, Oteiza PI, Fraga CG (2009). Cocoa, chocolate, and cardiovascular disease. *Journal of Cardiovascular Pharmacology*.

[88] Grassi D, Necozione S, Lippi C (2005). Cocoa reduces blood pressure and insulin resistance and improves endothelium-dependent vasodilation in hypertensives. *Hypertension*.

[89] Grassi D, Lippi C, Necozione S, Desideri G, Ferri C (2005). Short-term administration of dark chocolate is followed by a significant increase in insulin sensitivity and a decrease in blood pressure in healthy persons. *American Journal of Clinical Nutrition*.

[90] Baba S, Natsume M, Yasuda A (2007). Plasma LDL and HDL cholesterol and oxidized LDL concentrations are altered in normo- and hypercholesterolemic humans after intake of different levels of cocoa powder. *Journal of Nutrition*.

[91] Christensen B, Mosdol A, Retterstol L, Landaas S, Thelle DS (2001). Abstention from filtered coffee reduces the concentrations of plasma homocysteine and serum cholesterol—a randomized controlled trial. *American Journal of Clinical Nutrition*.

[92] Panagiotakos DB, Pitsavos C, Chrysohoou C, Kokkinos P, Toutouzas P, Stefanadis C (2003). The J-shaped effect of coffee consumption on the risk of developing acute coronary syndromes: the CARDIO2000 case-control study. *Journal of Nutrition*.

[93] Bidel S, Hu G, Qiao Q, Jousilahti P, Antikainen R, Tuomilehto J (2006). Coffee consumption and risk of total and cardiovascular mortality among patients with type 2 diabetes. *Diabetologia*.

[94] Greenberg JA, Dunbar CC, Schnoll R, Kokolis R, Kokolis S, Kassotis J (2007). Caffeinated beverage intake and the risk of heart disease mortality in the elderly: a prospective analysis. *American Journal of Clinical Nutrition*.

[95] Sumpio BE, Cordova AC, Berke-Schlessel DW, Qin F, Chen QH (2006). Green tea, the “Asian Paradox,” and cardiovascular disease. *Journal of the American College of Surgeons*.

[96] Peters U, Poole C, Arab L (2001). Does tea affect cardiovascular disease? A meta-analysis. *American Journal of Epidemiology*.

[97] Prior RL, Cao G (2000). Antioxidant phytochemicals in fruits and vegetables: diet and health implications. *HortScience*.

[98] Omenn GS, Goodman GE, Thornquist MD (1996). Effects of a combination of beta carotene and vitamin A on lung cancer and cardiovascular disease. *New England Journal of Medicine*.

[99] Davidson MH, Maki KC, Dicklin MR (2009). Effects of consumption of pomegranate juice on carotid intima-media thickness in men and women at moderate risk for coronary heart disease. *American Journal of Cardiology*.

[100] Geleijnse JM, Launer LJ, Van Der Kuip DAM, Hofman A, Witteman JCM (2002). Inverse association of tea and flavonoid intakes with incident myocardial infarction: the Rotterdam study. *American Journal of Clinical Nutrition*.

[101] Hertog MGL, Hollman PCH, Katan MB, Kromhout D (1993). Intake of potentially anticarcinogenic flavonoids and their determinants in adults in The Netherlands. *Nutrition and Cancer*.

[102] Knekt P, Järvinen R, Reunanen A, Maatela J (1996). Flavonoid intake and coronary mortality in Finland: a cohort study. *British Medical Journal*.

[103] Mukamal KJ, Maclure M, Muller JE, Sherwood JB, Mittleman MA (2002). Tea consumption and mortality after acute myocardial infarction. *Circulation*.

[104] Lichtenstein AH (1998). Soy protein, isoflavones and cardiovascular disease risk. *Journal of Nutrition*.

[105] Mink PJ, Scrafford CG, Barraj LM (2007). Flavonoid intake and cardiovascular disease mortality: a prospective study in postmenopausal women. *American Journal of Clinical Nutrition*.

[106] Jiang F, Dusting GJ (2003). Natural phenolic compounds as cardiovascular therapeutics: potential role of their antiinflammatory effects. *Current Vascular Pharmacology*.

[107] García-Lafuente A, Guillamón E, Villares A, Rostagno MA, Martínez JA (2009). Flavonoids as anti-inflammatory agents: implications in cancer and cardiovascular disease. *Inflammation Research*.

[108] Hooper L, Kroon PA, Rimm EB (2008). Flavonoids, flavonoid-rich foods, and cardiovascular risk: a meta-analysis of randomized controlled trials. *American Journal of Clinical Nutrition*.

[109] Berger A, Jones PJH, Abumweis SS (2004). Plant sterols: factors affecting their efficacy and safety as functional food ingredients. *Lipids in Health and Disease*.

[110] Hicks KB, Moreau RA (2001). Phytosterols and phytostanols: functional food cholesterol busters. *Food Technology*.

[111] Normén L, Holmes D, Frohlich J (2005). Plant sterols and their role in combined use with statins for lipid lowering. *Current Opinion in Investigational Drugs*.

[112] Demonty I, Ras RT, Van Der Knaap HCM (2009). Continuous dose-response relationship of the LDL-cholesterol-lowering effect of phytosterol intake. *Journal of Nutrition*.

[113] Quílez J, Rafecas M, Brufau G (2003). Bakery products enriched with phytosterol esters, *α*-tocopherol and *β*-carotene decrease plasma LDL-cholesterol and maintain plasma *β*-carotene concentrations in normocholesterolemic men and women. *Journal of Nutrition*.

[114] Wolfs M, de Jong N, Ocké MC, Verhagen H, Monique Verschuren WM (2006). Effectiveness of customary use of phytosterol/-stanol enriched margarines on blood cholesterol lowering. *Food and Chemical Toxicology*.

[115] Aguirre R, May JM (2008). Inflammation in the vascular bed: importance of vitamin C. *Pharmacology and Therapeutics*.

[116] Knekt P, Ritz J, Pereira MA (2004). Antioxidant vitamins and coronary heart disease risk: a pooled analysis of 9 cohorts. *American Journal of Clinical Nutrition*.

[117] Ye Z, Song H (2008). Antioxidant vitamins intake and the risk of coronary heart disease: meta-analysis of cohort studies. *European Journal of Cardiovascular Prevention and Rehabilitation*.

[118] Cook NR, Albert CM, Gaziano JM (2007). A randomized factorial trial of vitamins C and E and beta carotene in the secondary prevention of cardiovascular events in women: results from the women’s antioxidant cardiovascular study. *Archives of Internal Medicine*.

[119] Sesso HD, Buring JE, Christen WG (2008). Vitamins E and C in the prevention of cardiovascular disease in men: the physicians’ health study II randomized controlled trial. *JAMA - Journal of the American Medical Association*.

[120] Gaziano JM, Hennekens CH (1993). The role of beta-carotene in the prevention of cardiovascular disease. *Annals of the New York Academy of Sciences*.

[121] Krinsky NI, Johnson EJ (2005). Carotenoid actions and their relation to health and disease. *Molecular Aspects of Medicine*.

[122] Palace VP, Khaper N, Qin Q, Singal PK (1999). Antioxidant potentials of vitamin A and carotenoids and their relevance to heart disease. *Free Radical Biology and Medicine*.

[123] Engelhard YN, Gazer B, Paran E (2006). Natural antioxidants from tomato extract reduce blood pressure in patients with grade-1 hypertension: a double-blind, placebo-controlled pilot study. *American Heart Journal*.

[124] Liu S, Lee IM, Ajani U, Cole SR, Buring JE, Manson JE (2001). Intake of vegetables rich in carotenoids and risk of coronary heart disease in men: the physicians’ health study. *International Journal of Epidemiology*.

[125] Agarwal S, Rao AV (2000). Tomato lycopene and its role in human health and chronic diseases. *Canadian Medical Association Journal*.

[126] Osganian SK, Stampfer MJ, Rimm E (2003). Vitamin C and risk of coronary heart disease in women. *Journal of the American College of Cardiology*.

[127] Vivekananthan DP, Penn MS, Sapp SK, Hsu A, Topol EJ (2003). Use of antioxidant vitamins for the prevention of cardiovascular disease: meta-analysis of randomised trials. *Lancet*.

[128] Singh U, Devaraj S, Jialal I (2005). Vitamin E, oxidative stress, and inflammation. *Annual Review of Nutrition*.

[129] Gey KF, Puska P, Jordan P, Moser UK (1991). Inverse correlation between plasma vitamin E and mortality from ischemic heart disease in cross-cultural epidemiology. *American Journal of Clinical Nutrition*.

[130] Kushi LH, Folsom AR, Prineas RJ, Mink PJ, Wu Y, Bostick RM (1996). Dietary antioxidant vitamins and death from coronary heart disease in postmenopausal women. *New England Journal of Medicine*.

[131] Bolton-Smith C, Woodward M, Tunstall-Pedoe H (1992). The Scottish Heart Health Study. Dietary intake by food frequency questionnaire and odds ratios for coronary heart disease risk—II. The antioxidant vitamins and fibre. *European Journal of Clinical Nutrition*.

[132] Eidelman RS, Hollar D, Hebert PR, Lamas GA, Hennekens CH (2004). Randomized trials of vitamin E in the treatment and prevention of cardiovascular disease. *Archives of Internal Medicine*.

[133] Shekelle PG, Morton SC, Jungvig LK (2004). Effect of supplemental vitamin E for the prevention and treatment of cardiovascular disease. *Journal of General Internal Medicine*.

[134] Kalra EK (2003). Nutraceutical—definition and introduction. *AAPS PharmSci*.

[135] Osganian SK, Stampfer MJ, Rimm E, Spiegelman D, Manson JE, Willett WC (2003). Dietary carotenoids and risk of coronary artery disease in women. *American Journal of Clinical Nutrition*.

[136] Brigelius-Flohé R, Kluth D, Banning A (2005). Is there a future for antioxidants in atherogenesis?. *Molecular Nutrition and Food Research*.

[137] Stephens NG, Parsons A, Schofield PM (1996). Randomised controlled trial of vitamin E in patients with coronary disease: Cambridge Heart Antioxidant Study (CHAOS). *Lancet*.

[138] Blumberg JB, Frei B (2007). Why clinical trials of vitamin E and cardiovascular diseases may be fatally flawed. Commentary on “The relationship between dose of vitamin E and suppression of oxidative stress in humans”. *Free Radical Biology and Medicine*.

[139] Ornish D, Scherwitz LW, Billings JH (1998). Intensive lifestyle changes for reversal of coronary heart disease. *Journal of the American Medical Association*.

[140] Kushi LH, Lenart EB, Willett WC (1995). Health implications of Mediterranean diets in light of contemporary knowledge—1. Plant foods and dairy products. *American Journal of Clinical Nutrition*.

[141] De Lorgeril M, Salen P, Martin JL, Monjaud I, Delaye J, Mamelle N (1999). Mediterranean diet, traditional risk factors, and the rate of cardiovascular complications after myocardial infarction: final report of the Lyon Diet Heart Study. *Circulation*.

[142] Esposito K, Ciotola M, Giugliano D (2006). Mediterranean diet, endothelial function and vascular inflammatory markers. *Public Health Nutrition*.

[143] Riccardi G, Capaldo B, Vaccaro O (2005). Functional foods in the management of obesity and type 2 diabetes. *Current Opinion in Clinical Nutrition and Metabolic Care*.

[144] Samman S, Sivarajah G, Man JC, Ahmad ZI, Petocz P, Caterson ID (2003). A mixed fruit and vegetable concentrate increases plasma antioxidant vitamins and folate and lowers plasma homocysteine in men. *Journal of Nutrition*.

[145] Psaltopoulou T, Naska A, Orfanos P, Trichopoulos D, Mountokalakis T, Trichopoulou A (2004). Olive oil, the Mediterranean diet, and arterial blood pressure: the Greek European Prospective Investigation into Cancer and Nutrition (EPIC) study. *The American Journal of Clinical Nutrition*.

[146] Sofi F, Cesari F, Abbate R, Gensini GF, Casini A (2008). Adherence to Mediterranean diet and health status: meta-analysis. *British Medical Journal*.

[147] Moore TJ, Conlin PR, Ard J, Svetkey LP (2001). DASH (Dietary Approaches to Stop Hypertension) diet is effective treatment for stage 1 isolated systolic hypertension. *Hypertension*.

[148] Blumenthal JA, Babyak MA, Sherwood A (2010). Effects of the dietary approaches to stop hypertension diet alone and in combination with exercise and caloric restriction on insulin sensitivity and lipids. *Hypertension*.

[149] Fung TT, Chiuve SE, McCullough ML, Rexrode KM, Logroscino G, Hu FB (2008). Adherence to a DASH-style diet and risk of coronary heart disease and stroke in women. *Archives of Internal Medicine*.

[150] Parikh A, Lipsitz SR, Natarajan S (2009). Association between a DASH-like diet and mortality in adults with hypertension: findings from a population-based follow-up study. *American Journal of Hypertension*.

[151] Obarzanek E, Sacks FM, Vollmer WM (2001). Effects on blood lipids of a blood pressure-lowering diet. The Dietary Approaches to Stop Hypertension (DASH) Trial. *American Journal of Clinical Nutrition*.

[152] Appel LJ, Miller ER, Jee SH (2000). Effect of dietary patterns on serum homocysteine: results of a randomized, controlled feeding study. *Circulation*.

[153] DeWitt Goodman S, Hulley SB, Clark LT (1988). Report of the National Cholesterol Education Program Expert Panel on detection, evaluation, and treatment of high blood cholesterol in adults. *Archives of Internal Medicine*.

[154] Jenkins DJA, Kendall CWC, Faulkner D (2002). A dietary portfolio approach to cholesterol reduction: combined effects of plant sterols, vegetable proteins, and viscous fibers in hypercholesterolemia. *Metabolism*.

[155] Ridker PM, Hennekens CH, Buring JE, Rifai N (2000). C-reactive protein and other markers of inflammation in the prediction of cardiovascular disease in women. *New England Journal of Medicine*.

[156] Key TJ, Fraser GE, Thorogood M (1998). Mortality in vegetarians and non-vegetarians: a collaborative analysis of 8300 deaths among 76,000 men and women in five prospective studies. *Public Health Nutrition*.

[157] Willcox BJ, Willcox DC, Todoriki H (2007). Caloric restriction, the traditional okinawan diet, and healthy aging: the diet of the world’s longest-lived people and its potential impact on morbidity and life span. *Annals of the New York Academy of Sciences*.

[158] Suzuki M, Wilcox BJ, Wilcox CD (2001). Implications from and for food cultures for cardiovascular disease: longevity. *Asia Pacific Journal of Clinical Nutrition*.

[159] Willcox DC, Willcox BJ, Todoriki H, Curb JD, Suzuki M (2006). Caloric restriction and human longevity: what can we learn from the Okinawans?. *Biogerontology*.

[160] Perez-Jimenez J, Saura-Calixto F (2008). Grape products and cardiovascular disease risk factors. *Nutrition Research Reviews*.

[161] Jensen MK, Koh-Banerjee P, Hu FB (2004). Intakes of whole grains, bran, and germ and the risk of coronary heart disease in men. *American Journal of Clinical Nutrition*.

